# Hyperspectral Image Enhancement and Mixture Deep-Learning Classification of Corneal Epithelium Injuries

**DOI:** 10.3390/s17112644

**Published:** 2017-11-16

**Authors:** Siti Salwa Md Noor, Kaleena Michael, Stephen Marshall, Jinchang Ren

**Affiliations:** 1 Centre of Excellent Signal and Image Processing, Department of Electronic and Electrical Engineering, University of Strathclyde, Glasgow G1 1XW, UK; siti-salwa-binti-md-noor@strath.ac.uk (S.S.M.N.); jinchang.ren@strath.ac.uk (J.R.); 2 Glasgow Centre for Ophthalmic Research, Gartnavel General Hospital, Glasgow G12 0YN, UK; kaleena.michael@gmail.com

**Keywords:** corneal epithelium, hyperspectral imaging, support vector machine, convolutional neural networks, image enhancement

## Abstract

In our preliminary study, the reflectance signatures obtained from hyperspectral imaging (HSI) of normal and abnormal corneal epithelium tissues of porcine show similar morphology with subtle differences. Here we present image enhancement algorithms that can be used to improve the interpretability of data into clinically relevant information to facilitate diagnostics. A total of 25 corneal epithelium images without the application of eye staining were used. Three image feature extraction approaches were applied for image classification: (i) image feature classification from histogram using a support vector machine with a Gaussian radial basis function (SVM-GRBF); (ii) physical image feature classification using deep-learning Convolutional Neural Networks (CNNs) only; and (iii) the combined classification of CNNs and SVM-Linear. The performance results indicate that our chosen image features from the histogram and length-scale parameter were able to classify with up to 100% accuracy; particularly, at CNNs and CNNs-SVM, by employing 80% of the data sample for training and 20% for testing. Thus, in the assessment of corneal epithelium injuries, HSI has high potential as a method that could surpass current technologies regarding speed, objectivity, and reliability.

## 1. Introduction

Visual impairment and blindness can occur as a result of various circumstances, which can broadly be categorised into infectious and non-infectious causes [1]. It is estimated that about 285 million people worldwide are either visually impaired or blind, and approximately 80% of these are thought to be due to preventable causes [1]. Blindness inflicted by diseases of the cornea, which is the outermost layer of the eye, plays a significant role in these statistics, second only to cataracts in overall importance [2].

The diagnosis of corneal diseases can pose a challenge, even amongst eye specialists. The advance of new assistive tools to aid both the specialist and non-specialist is an essential step towards reducing the problem of blindness worldwide. Hyperspectral imaging is a relatively new yet advancing technology that combines imaging with spectroscopy, which has made a gradual change in biomedical applications. Initially developed for use in Earth remote sensing [3], the technology underwent major advances to conform to different challenges across various industries, including space exploration [4], food safety and quality control [5,6,7,8,9], archaeology for conservation and authentication [10], and more recently, in the area of healthcare in clinical diagnostics and surgical guidance.

An excellent example of the viability of this technology in biomedicine is HELICoiD, a European collaborative project, co-funded by the European Union, established to support hyperspectral imaging technology in real time cancer detection of malignant primary brain tumours during surgery [11]. HSI has also been adopted at a research level in quantifying degrees of skin burns [12], with the added potential of providing clinicians with useful information in monitoring the healing process during treatment. Surgically, HSI has been trialled to assist visualisation during surgery by enhancing tissue visibility [13,14], as well as the local detection of pathological tissues, without the need for invasive tissue biopsies [14,15].

## 2. Related Works

When designing a device for the assessment of the human eye, it is advantageous for it to be non-invasive, user-friendly, and contactless. These features often influence its role in clinical use. Below are brief descriptions of assistive diagnostic tools commonly employed in a clinical setting for the examination of the cornea.

Ophthalmologists widely use a slit lamp that combines microscopy with different illumination techniques for a detailed examination of the eye. Gulstrand is credited with this invention in 1911 [16], although the device has subsequently undergone numerous advances and modifications over the decades. The modern day slit lamp enables the ophthalmologist to examine the living eye and is equipped with various adjustable controls for alteration of magnification, level and angle of illumination, beam width and height with multiple light filters. Despite this versatility, its limitations lie in its inability to provide objective measures in the presence of pathology.

Aspects of eye examination (adjuncts to slit lamp examination) such as the ultrasound pachymetry [17] for measurements of corneal thickness, gonioscopy [18] for evaluation of drainage angles, and tonometry [19] for intraocular measurements, often require prolonged contact with the patient’s eye and can be poorly tolerated in the paediatric group and among some adults.

As a result, there is a push in the bioengineering [20] sector to develop technology that could avoid physical contact and improve patient tolerability. Some examples include specular microscopy [21] for corneal thickness measurements and endothelial layer analysis, Schiempflug imaging [22] technology combined with the pentacam [23] allows the cross-section of the anterior segment to be visualised and measured as well of corneal curvature to be mapped topographically for refractive surgical planning.

In recent years, Optical Coherence Tomography (OCT) [24] has been employed for routine clinical use due to its ability to produce accurate B-scan images of the posterior and more recently the anterior segment of the eye. Infrared light of wavelength 800 nm and 1310 nm [25] is used to allow precise and detailed cross-sectional inspection of ocular tissues in the anterior and posterior segment, respectively.

The role of hyperspectral imaging on the eye has been explored in the literature, although many approaches currently remain in the research domain. Reynaud et al. [26] studied the spectral response on the rabbit cornea using hyperspectral imaging interfaced with the confocal microscope, and were able to isolate individual cells and structures based on their spectral signatures. They found that corneal stroma and the endothelial layer generated a specific spectral response in the range 440 nm to 730 nm [26]. No other hyperspectral imaging related work on the porcine cornea has been published to our knowledge. Attempts to employ hyperspectral imaging technology for assessment of the posterior segment of the eye (retina) has seen more progress, particularly in measuring relative changes in oxygen saturation of the retina [27], as well as abnormalities in oxygen saturation in the optic nerve head of early glaucoma sufferers [28]. Li et al. [29] performed a study on 40 healthy Wistar rats, divided into normal control, diabetic, and erythropoietin (EPO) groups. Upon examination of the retinae, their team found the inner and outer nuclear and inner plexiform layers to be distinguishable using various spectral bands.

The study of corneal pathologies, attempts to quantify and assess the cornea objectively, and are seeing encouraging progress through different technologies such as OCT, which can produce detailed cross-sectional tissue information, but not in front surface view. Fukuda and Sasaki [30] attempted to quantify corneal epithelium injury by measuring electrical corneal resistance with some success.

In summary, various solutions have been offered in the pursuit of an ideal and robust tool for corneal assessments, with some related examples discussed briefly above. Here we provide an alternative way by combining hyperspectral imaging with image processing analysis. The objectives of this study are to (1) investigate the ability of a hyperspectral device to extract data from corneal epithelium tissues by analysing spectral signatures; (2) predict the potential for clinical diagnostics, by simplifying the clinician's methods of examination, in detecting corneal epithelium injuries; (3) visualise and analyse the spatial and spectral features; and (4) classify injured and healthy corneal epithelium using an SVM with GRBF kernel and CNNs.

**Figure 1:**
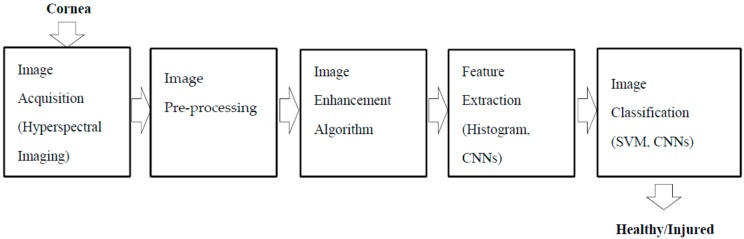
Corneal epithelium appraisal using hyperspectral imaging and image processing analysis.

The significant contribution in this paper is the fusion of hyperspectral imaging with image process analysis (Figure 1) as a way to appraise and visualise the cornea/injury in detail, particularly the corneal epithelium without eye fluorescein stain.

## 3. Materials and Methods

### 3.1. Experimental Set-Up

**Figure 2:**
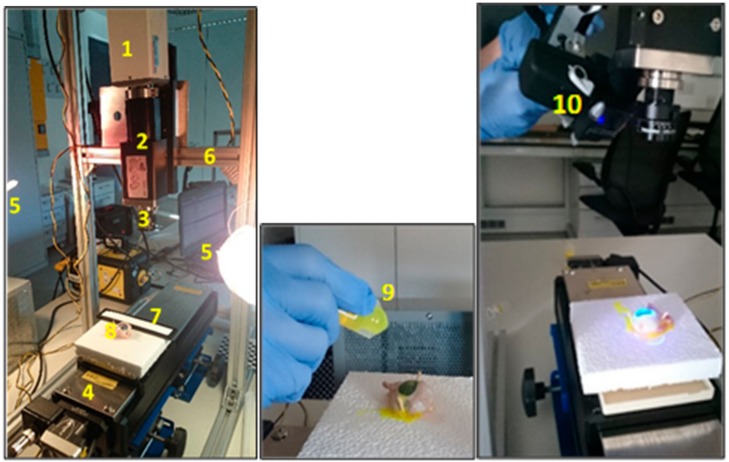
Column 1: Experimental setup. (1) CCD Camera; (2) Spectrograph; (3) Lens; (4) Translation stage; (5) Left and right halogen lamp; (6) Frame; (7) Spectralon/white panel; (8) Sample/porcine eye, Column 2: (9) Staining process, Column 3: (10) Blue lamp illumination source for stained eye.

Hyperspectral image acquisition is performed through the HSI system as illustrated in Figure 2. The line scanning also known as the pushbroom [31] method was used for image collection in the series of experiments, detailed in Table 1.

The porcine eye is anatomically and biochemically similar to the human eye and is a common alternative used in wet lab-based research and surgical training [32,33]. Corneal abrasion resulting in partial loss of the outermost corneal layer (called the epithelium) was chosen as the clinical problem to be studied. The loss of the epithelial layer is frequently undetected by assessors, and is often only visible when the corneal surface has been treated with diluted 1% fluorescein drops and viewed under cobalt blue lighting. This is possible because the abraded areas of the cornea retain the dye and fluoresce brightly in cobalt blue light.

During this work, all ethical obligations were complied with, and the lab work sessions were carried out according to the rules set out by the governing organisations. All porcine eyes were resourced as by-products of the food industry. The details about sample preparation used have been previously reported [34].

#### 3.1.1. Hyperspectral Cornea Image Collections

**Figure 3:**
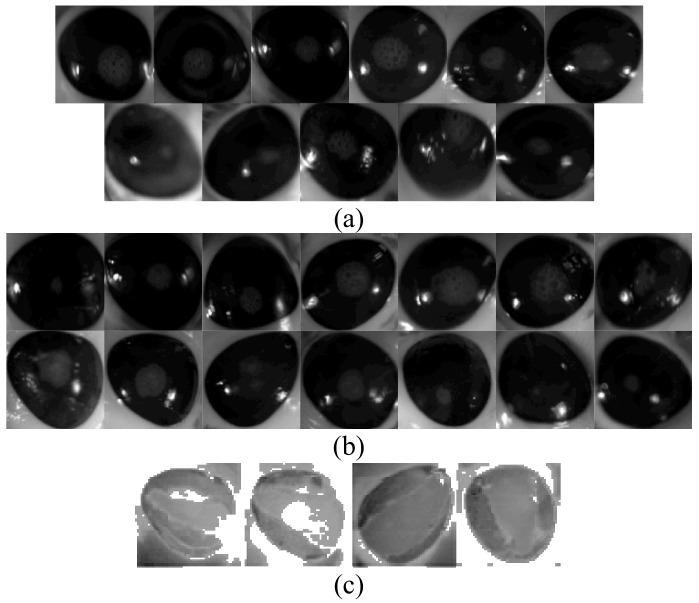
Twenty five hyperspectral images sliced at band-100. All images were normalised and resized to 100 by 100 pixels. (**a**) Rows 1 and 2 are images of healthy corneas; (**b**) Rows 3 and 4 are images of corneas with induced epithelial injuries; (**c**) Row 5 are ground truth images.

A total of 25 hyperspectral images are shown in Figure 3 and consist of the following: 14 with corneal epithelium injury (abnormal), and 11 with completely intact corneal epithelium (normal). All eyes were included for further analysis in this work.

All 25 eyes were scanned without any fluorescein staining. Of the 14 eyes with abnormal epithelium, four eyes were chosen at random for application of fluorescein stain and repeated scanning. Images from the stained eyes formed the control group (ground truth images).

### 3.2. Image Enhancement

Image enhancement is a process that allows for the transformation of an original image when contrast is insufficient, or when the image has a high level of noise to be converted to an image that can be utilised for further analysis [35]. The methods used for enhancement vary according to the chosen imaging modality. For example, the methods used to enhance MRI images are unlikely to represent the best approach to enhance hyperspectral images taken in the visible near infrared band of the electromagnetic spectrum [36].

There is no universal enhancement algorithm that is effective for all types of images. The ultimate goal of enhancement algorithms is to increase the contrast between structures of interest and their surroundings, as well as to reduce noise. In addition, enhancement also improves and refines image segmentation, especially in images where the distinction between normal and abnormal tissue is unclear, for human interpretation as well as automatic systems [35,37]. The following section describes the HSI image pre-processing and enhancement applied in this paper.

#### 3.2.1. HSI Data Normalisation

An essential step in HSI imaging, before image acquisition, is a flat-field correction for data normalisation. A white balance and dark current measurements [38] were used to acquire relative reflectance from the sample. The dark current of the sensor was recorded with the sensor being protected from incoming light. This step is required to measure the actual dynamic range of the sensor. Together with the white balance step, both measures were also used to identify corrupted or defected pixels in the pushbroom sensor of the hyperspectral camera. The white balance material was calibrated at regular intervals by comparing its reflectance properties with those of a spectralon probe to compensate ageing or usage degradation of white balance quality. Data from black current and white balance measurements were used to correct the measured material image. The main purpose of this correction is to eliminate artefacts and noise effects on the sample [39], computed with the following equation [31]:

(1) $$Rs\left(\lambda \right)=\frac{Is\left(\lambda \right)-Id\left(\lambda \right)}{Ir\left(\lambda \right)-Id\left(\lambda \right)}\;\times \;100\text{\%}$$

where *Rs(λ)* is the relative reflectance of the sample object, *Is(λ)* is the sample or measured image, *Id(λ)* is the dark image acquired when the light is absent by closing the lens with cap, *Ir(λ)* is the image obtained from the spectralon white bar, and λ is the wavelength.

#### 3.2.2. Brightness and Contrast Adjustment 

Most of the captured images appeared relatively dark due to exposure attained during image acquisition by the hyperspectral imaging system. This low-contrast dark image requires brightness and contrast adjustment for better visibility of the image details. Gamma correction or power-law transformation, *s* = *r^γ^* is essential for contrast manipulation when the image is likely to be too dark [36]. The transformation can be obtained simply by varying the value of *γ*, according to the power-law curves, by setting *γ* > 1 to make an image darker, and vice versa.

#### 3.2.3. Morphological Transformation

The mathematical morphological (MM) technique is widely used in shape-based image processing for region segmentation, threshold processing, noise elimination, and hole filling [35]. MM is particularly useful in describing shapes using set theory by a structuring element (SE). Typically, SE is chosen with the same size and shape as the objects to be processed in the input image. For example, to find lines in an image, create a linear SE. There are two categories of SE in gray-scale morphology: flat and non-flat [36]. Flat is 2-dimensional and non-flat is 3-dimensional. SE consists of a matrix of 0s and 1s, typically much smaller than the image being processed. The origin, which is a centre of the SE, will identify the pixel of interest and define the neighbourhood used in the processing of each pixel.

These SEs are also considered in the primitive operations, namely erosion and dilation processing. The following explanation will focus on the erosion operator as it is used in this research. Erosion was applied to two sets of matrices: gray-level image matrix *A*(*x,y*), and the structural element matrix *B*(*u,v*). Erosion of *A* by *B* is the set of all points *z* in *B*, translated by *z*, is comprised of *A*, written *A*θ*B* = {*z*|*B_z_* ⊆ *A*} [36].

**Figure 4:**
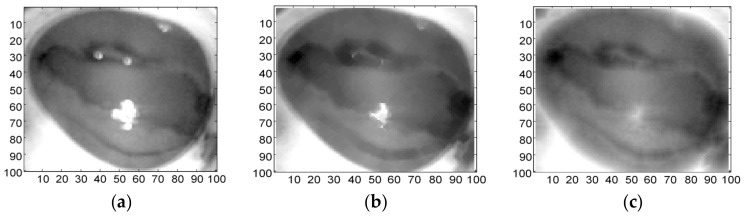
Eroded Image. (**a**) Original image; (**b**) Eroded with ‘disk’ SE; (**c**) Eroded with ‘spherical’ SE.

A spherical or ball shape non-flat SE was used to probe the image, which was constructed in 3D structure and consisted of the radius in the *x*-*y* plane and added *z* value to define the third dimension. Spherical SE was used with radial decomposition [40] to accelerate such operations as the top hat and rolling ball transformations [41]. This non-flat SE also improved the performance of morphological filtering in terms of the smooth opening and closing of electrocardiogram (ECG) signals [42]. Although disk SE is commonly used for medical images [43], it is unlikely to work well in this study (Figure 4b). In contrast, the spherical shape (Figure 4c) had removed the glare with preservation of vital image features (boundary of abnormal tissue). This is because it has been eroded by a spherical SE about the size of the glare. This glare must be removed from the image for further processing.

#### 3.2.4. Laplacian of Gaussian Filter (LoG)

One of the earliest edge detectors was introduced by Marr-Hildreth [44] and is also known as the Laplacian of Gaussian. LoG has the ability to detect boundaries or edges at different scales while dealing well with intensity changes from surface disruptions, reflectance, or illumination. The combination of a 2D Gaussian function (image smoothing), G = (*exp* − (*x*^2^ + *y*^2^/*2σ*^2^)), and a Laplacian operator (edge detection), ∇^2^ = (*d*^2^/*dx*^2^ + *d*^2^/*dy*^2^), gives the expression: ∇^2^G(*x,y*) = [*x*^2^ + *y*^2^ − *2σ*^2^/*σ*^4^] *exp* − (*x*^2^ + *y*^2^/*2σ*^2^) [36]. By applying LoG to hyperspectral images, a variety of images were generated, subject to alterations in its parameters. Larger values of sigma caused the edges to blur, while smaller values led to detailed and sharp detectable edges but prone to noise.

#### 3.2.5. Principal Component Analysis (PCA)

One of the issues with hyperspectral imaging is that it generates huge data sets, much of which are redundant. PCA [45] is a popular image transformation method that we have used to resolve this issue and provide uncorrelated data (transform high to low dimensional). Several principal components with maximum variability were selected for subsequent processing stages. This algorithm is well described previously in hyperspectral image classification [46]. The method includes mean subtraction, computation of covariance matrix, calculation of eigenvectors and eigenvalues, selection of components and forming feature vectors to derive a new data set.

In this work, PCA was applied to the hyperspectral images. The background of mathematical expression used was previously described [46,47]. Each image in spatial dimension *m* × *n* pixels was transformed into an image vector, consisting of spectral wavelength N-dimensional samples, into one image matrix *M*, *[ImgVec1:…: ImgVecN]*. The mean vector (2) for every image vector, *xi = [x1, x2, …, xN ]^T^* was computed and transformed into covariance matrix (3). The covariance matrix was then used to generate eigenvectors *(e1, …, en)* and corresponding eigenvalues *(λ1, …, λn)*. The eigenvectors were then arranged in higher to a lower order of eigenvalues to form the principal components that correspond to the number of hyperspectral bands. The mean vector and covariance matrix are computed as follows:

Mean vector:

(2) $$\bar{m}=\frac{1}{M}\;\overset{M}{\underset{i=1}{\sum }}{\left[x_{1},x_{2},\cdots ,x_{N}\right]}^{T}$$

Covariance matrix:

(3) $$Covx=\frac{1}{M}\;\overset{M}{\underset{i=1}{\sum }}\left(x_{i}-\bar{m}\right){\left(x_{i}-\bar{m}\right)}^{T}$$

where *M* is an image dimension, *x* is image pixel, and *T* denotes transpose operation.

#### 3.2.6. Image Subtraction

The image subtraction in this work was performed on images extracted from PCA. Let *g*(*x,y*) denote an image difference by the subtraction of PC1 *f*(*x,y*) from PC10 *h*(*x,y*) or vice versa; forming, *g*(*x,y*) = *f*(*x,y*) − *h*(*x,y*) or *h*(*x,y*) − *f*(*x,y*). The image differences were enhanced, with details previously described [36].

### 3.3. Support Vector Machine-Gaussian Radial Basis Function (SVM-GRBF)

SVM is preferred commonly used classifier for machine learning applications due to its capability to work with different types of kernel or covariance function [48] by dot product rule. Based on the 2D-image feature distribution obtained from the histogram, it was not possible to separate the two classes of image data by a linear transformation in input space. Therefore, a non-linear SVM classifier was employed with Gaussian radial basis function kernel, as its performance in hyperspectral remote sensing classification was better than either SVM-linear, K-NN classifier, or standalone of RBF classifier [49]. The linear and non-linear SVM is represented by Equations (4) and (5), respectively:

(4) $$f{\left(x\right)}_{linear}=\underset{i\in sv}{\sum }\alpha_{i}y_{i}\left(x_{i},x^{\prime }\right)+b$$

(5) $$f{\left(x\right)}_{non-linear}=\underset{i\in sv}{\sum }\alpha_{i}y_{i}K\left(x_{i},x^{\prime }\right)+b$$

where $\alpha_{i}y_{i}$ is a data point, $K\left(x_{i},x^{\prime }\right)$ is a kernel, and b is a bias.

Then the GRBF kernel which is represented as $K\left(x_{i},x^{\prime }\right)$ in Equation (5) is denoted as:

(6) $$K_{GRBF}\left(x_{i},x_{j}\right)=\exp\left(-\frac{{\left|x-x_{i}\right|}^{2}}{2\sigma^{2}}\right)$$

where σ is the width of the radial basis function, and different values of width will affect the boundary of classification between normal and abnormal classes.

Before training the model, data normalisation is carried out. This is to ensure that all attributes have the same importance. In this paper, each column of the feature vector in both the training and testing sets was normalised to a length of 1. The MATLAB function ‘normc’ was used for data normalisation to preserve the relationship between the vector components.

### 3.4. Convolutional Neural Networks (AlexNet)

**Figure 5:**
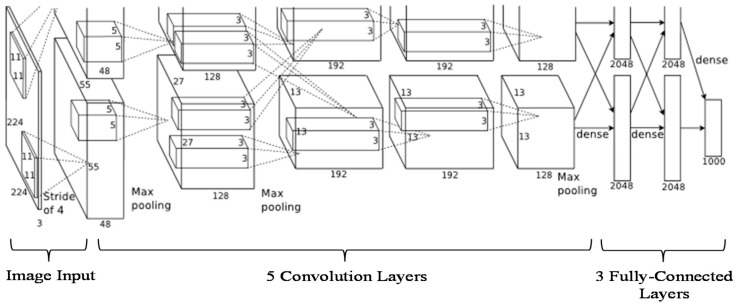
AlexNet Architecture.

A standard neural network known as AlexNet [50] consists of 1.2 million high-resolution images and can be used to classify 1000 different classes. It comprises millions of parameters, hundreds thousand neurons, five convolutional layers (some of which consist of max-pooling layers), and three fully-connected layers with a 1000-way SoftMax (Figure 5). The motivation of using AlexNet is that it has been used on small data for fingerprint detection [51] on a pretrained model with good results. Furthermore, AlexNet has been trained on rich feature representations for a wide range of images. In this paper, we have applied 25 images obtained from PC subtraction consisting of the normal and abnormal cornea. To enrich the training data, we employed image flipping and rotation for data augmentation in order to increase classification accuracy [51]. In total, we used 94 images after image augmentation for classification. All images were transformed into image vectors and randomly split into two sets for training and testing. The ratio of training to testing was varied across the following values: 0.1(10% for training, 90% for testing), 0.2, 0.3, 0.4, 0.5, 0.6, 0.7, 0.8, 0.9, and 1.0 in order to determine the optimal accuracy. The time consumed using a single CPU for every distribution was also recorded.

As AlexNet was designed to classify 1000 images, it not suitable (overfitting concerns) for use directly with very small data set and only two classes that we have. Therefore, we applied two approaches to classify the data into two classes, healthy and injured, by using transfer learning [52] and deep feature extraction [53,54] on a pretrained AlexNet model. These approaches explained as follows:

#### 3.4.1. Transfer Learning Using Pretrained AlexNet with a Fine-Tuned Model on the Cornea Images

The last three layers were configured for 1000 classes of the original trained network. In this work, these layers plus some others layers were fine-tuned (see Table 2, underlined-bold items) for the new classification cornea problem.

The input image was resized to 227 × 227 × 3, and the network was trained with a single CPU. The layers other than the last three were transferred directly (keeping the layer weights of the pretrained network) to the new classification task, whilst the final three layers were replaced with a fully connected layer, a softmax layer, and a classification output layer. The new fully connected layer was trained to classify the cornea images into just two classes. To increase the learning rate in the new layers, we set values for both the weight learn rate factor and the bias learn rate factor to 20, with the small initial learning rate to 0.001, and the number of epochs to five. Finally, the cornea images were trained in a network consisting of the transferred and the new layers. As a result, the validation images were classified using the fine-tuned network, and the accuracy was computed from the fraction of labels that the network correctly predicted.

#### 3.4.2. Feature Extraction with Pretrained AlexNet on Cornea Images

**Figure 6:**
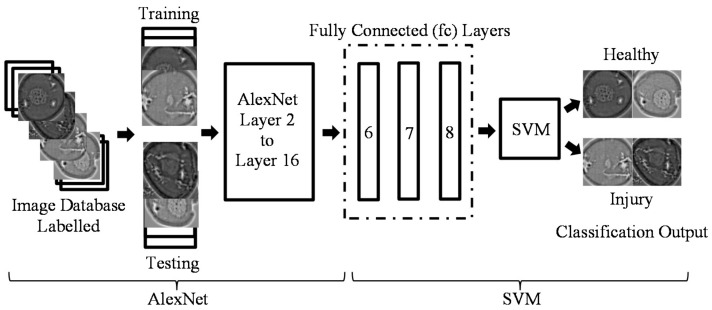
Feature extraction with pretrained AlexNet on cornea images classification using SVM.

Feature extraction is the easiest and fastest way exploit the representational power of pretrained deep networks. The network produces a hierarchical representation of input images. We used activations on the fully connected layer ‘fc6’ for feature extraction of the training and test images (Figure 6).

The class labels from the training and test data were extracted. Then, the features extracted from the training images were used as predictor variables and trained using linear support vector machine (SVM). The test images were classified using the trained SVM model based on the features extracted from the test images.

### 3.5. Mixture AlexNet and SVM-Linear

The fusion of AlexNet and SVM-linear classifier was used for comparison. Due to the complex architecture involved in AlexNet, the learning process can be very time-consuming. This disadvantage could potentially be resolved by the use of a graphics processing unit (GPU). However, GPU is less readily used or available, thereby limiting future applicability. Therefore, for central processing unit (CPU) users, the combination of AlexNet and SVM-linear denoted in (4) is more than sufficient, where AlexNet performs the high-level feature extraction while SVM-linear carried out the classification (Figure 6).

**Figure 7:**
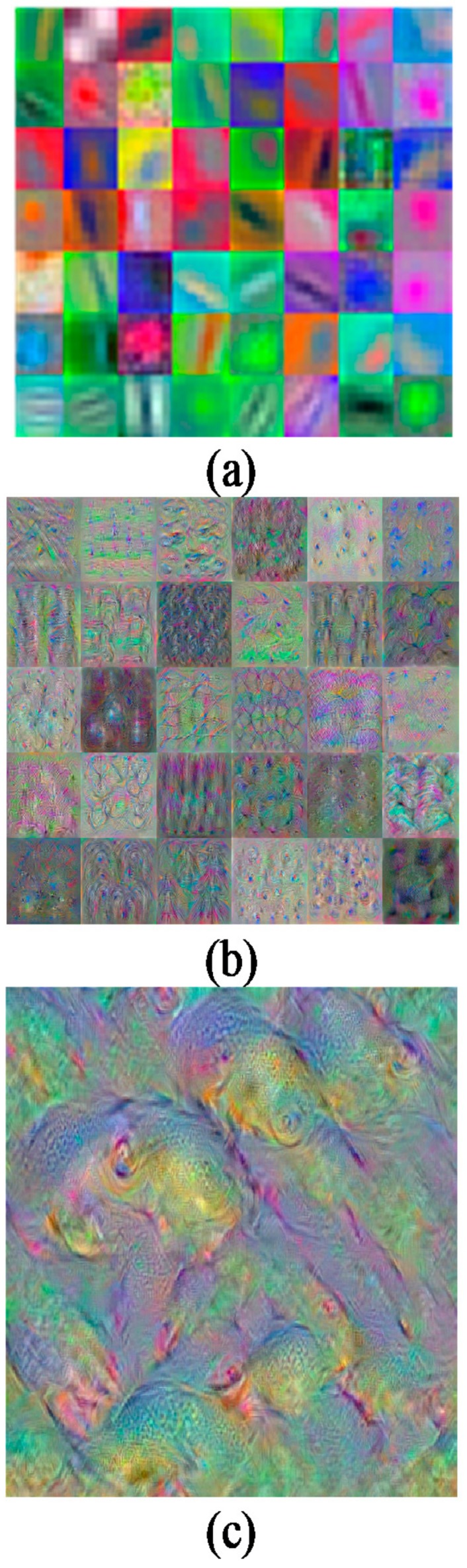
Sample of features extraction. (**a**) Conv1 (56 channels); (**b**) Conv5 (30 channels); and (**c**) FC8 layer (channel 1).

Figure 7 depicts samples of features extracted from convolution 1, convolution 5, and fully-connected 8 (FC8). There are three possible layers of feature extraction output in AlexNet, namely FC6 (layer 17), FC7 (layer 20), and FC8 (layer 23) consisting of 4096, 4096, and 1000 feature dimensions respectively. Any one of these three layers can be used as feature representation entries to the SVM classifier. In this combination, convolutional layers were used to learn a better representation of the input image, and SVM classification was performed on the fc output during training and testing with such automatically extracted features. This is the reason AlexNet-SVM runs much faster than a standalone AlexNet.

## 4. Results and Discussion

**Figure 8:**
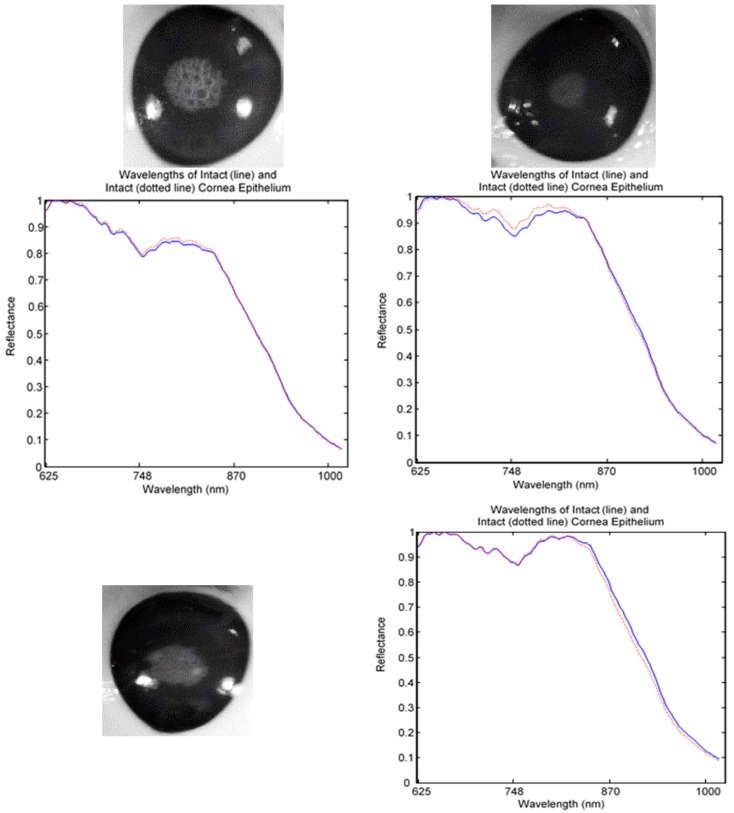
Reflectance signatures of healthy eye.

**Figure 9:**
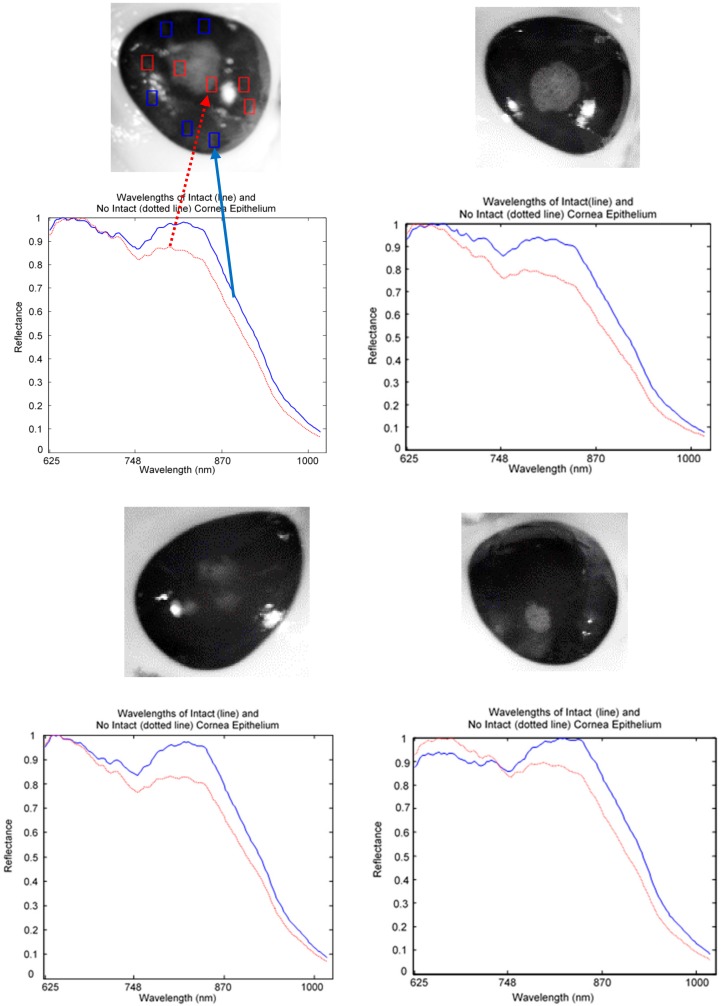
Reflectance signature of injured eye without stain.

**Figure 10:**
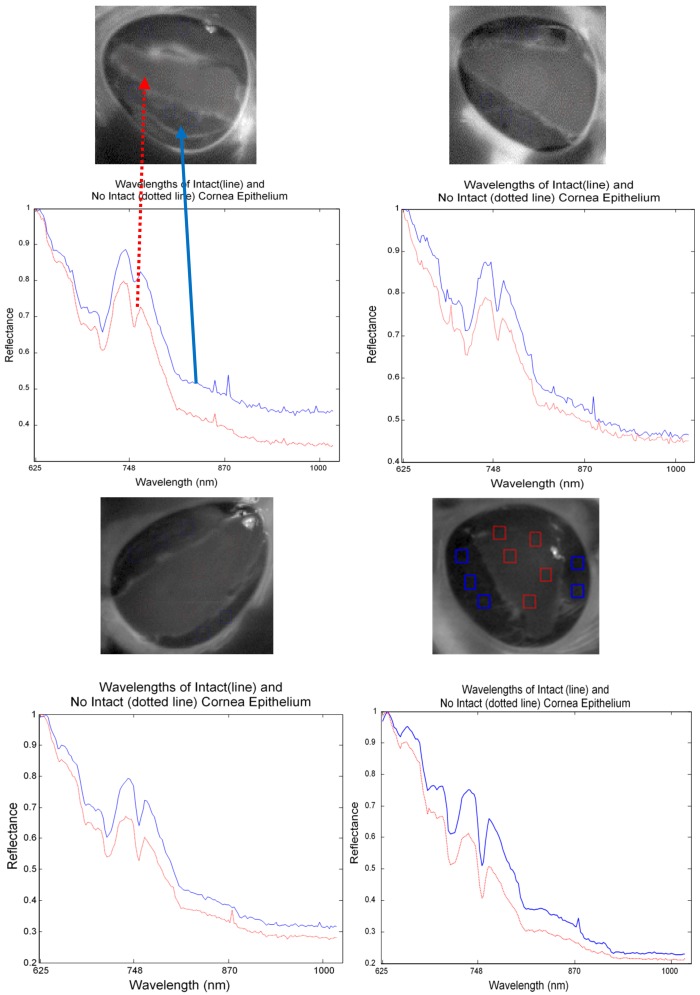
Reflectance signature of injured eye with stain (control image).

The captured HSI images were divided into healthy, injured, and injured eye with stain as a control image for analysis. For spectral analysis, ten squares size 5 × 5 were randomly cropped from each image. Five of the squares were used to represent normal (line) and another five were used for abnormal (dotted line) tissues. The mean of the reflectance was then plotted to gain the spectral signature. Figure 8 shows a mean reflectance signature for a healthy eye: there was no difference in spectral signatures taken from different locations. In contrast, Figure 9 and Figure 10 show the difference between normal and abnormal tissues from different injured eyes.

The image pre-processing and enhancement described in Section 3.2 were chosen to improve distinction and visualisation of normal and abnormal corneal epithelium.

**Figure 11:**
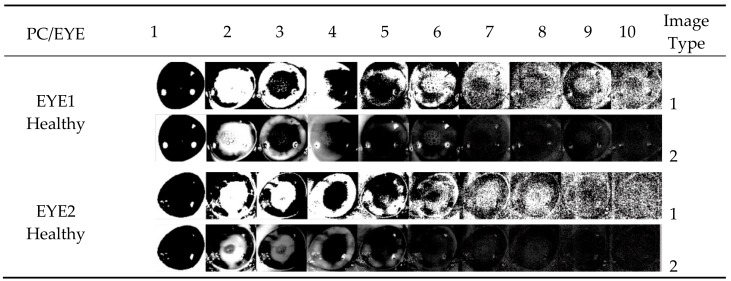

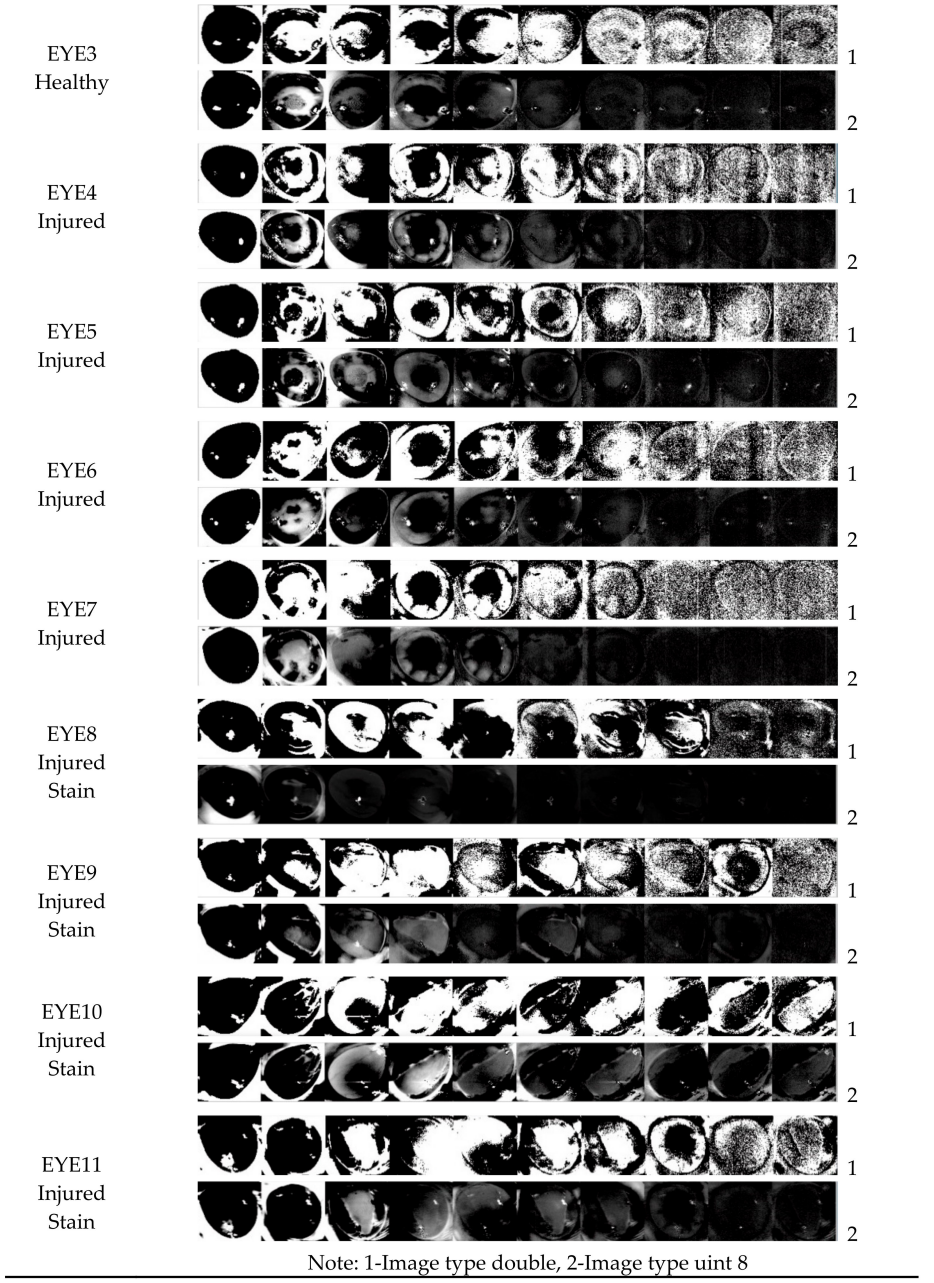
Ten principal components (PC) of eleven eyes transformed with PCA. The clinical information appears in several PC for EYE8 until EYE11 as these images were stained. In contrast, EYE1 to EYE7 which were without stained appear similar even though EYE4 to EYE7 had abnormal corneal epithelium.

In Figure 11, eleven eyes were transformed using PCA before using image enhancement. It is shown that all the eyes which were without stained appear similar between normal and abnormal cornea. In contrast, all the eyes which were stained gave clear clinical information from the injury area. The image enhancement algorithms for the hyperspectral image of the porcine cornea can be summarised as follows:

*Step 1*. A full band of a hyperspectral image was loaded, Input = M*N*λ.

**Figure 12:**
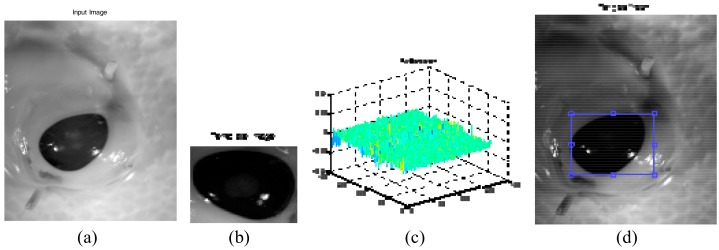
Template matching-FFT based correlation. (**a**) input image; (**b**) template image; (**c**) correlation plot, and (**d**) template matched.

*Step 2*. Select a region of interest (cornea) using template matching method (FFT-based correlation), see Figure 12.

*Step 3*. The image was resized to 100 by 100, *A* ϵ R^M^*^N^*^λ^.

*Step 4*. Contrast transformation was applied to all selected bands (i.e., band 50 to band 100).

Parameter setting: clip pixel level, and gamma.

**Figure 13:**
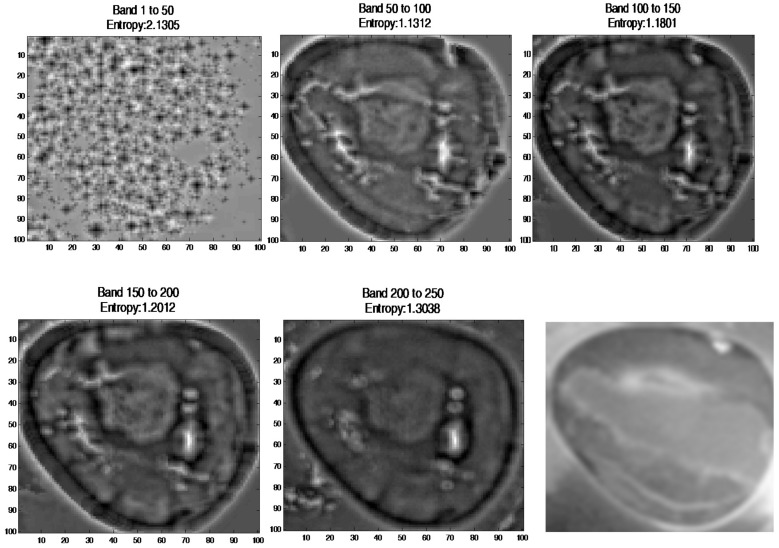
Output image at band 1 to 50, 50 to 100, 100 to 150, 150 to 200, and 200 to 250. Image at row 2 column 3 is a ground truth image.

The bands were selected using spatial entropy based mutual information [55] of the spectral image in range 0 to 50,50 to 100, 100 to 150, 150 to 200, and 200 to 250. As a result, the image at wavelength 503 nm to 625 nm was selected for further processing (Figure 13).

*Step 5*. Morphology operation using erosion was applied to all selected bands.

Parameter setting: structure element (SE) ‘ball’size.

*Step 6*. Image filtering using Laplacian of Gaussian (LoG) was performed on all selected bands.

Parameter setting: filter size, and sigma.

**Figure 14:**
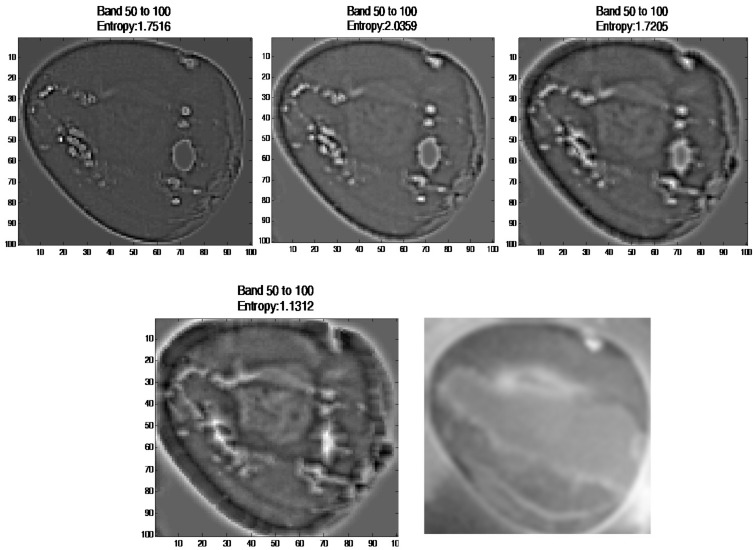
Output image at four different parameter sets ([5,5], [3 3], 0.1), ([15,15], [5 5], 0.1), ([25,25], [7 7], 0.1), and ([50,50], [9 9], 0.1). Image at row 2 column 2 is a ground truth image.

The three-parameter setting (SE size, filter size, sigma) was selected based on visualise the spectral image with four different parameter sets ([5,5], [3 3], 0.1), ([15,15], [5 5], 0.1), ([25,25], [7 7], 0.1), and ([50,50], [9 9], 0.1). As a result, the image with parameters ([50,50], [9 9], 0.1) showed the injured boundary (Figure 14).

*Step 7*. Principal component analysis of all selected bands was computed, display 10 PCs only which contains almost 100% of the variance. PCs with zero variance were neglected.

**Figure 15:**
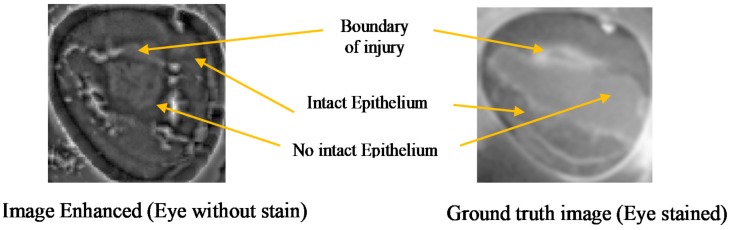
Image enhanced and ground truth image.

*Step 8*. Image subtraction was performed between PC2 (2nd largest variance) and PC1

(1st largest variance). Output result after enhancement is illustrated in Figure 15.

**Figure 16:**
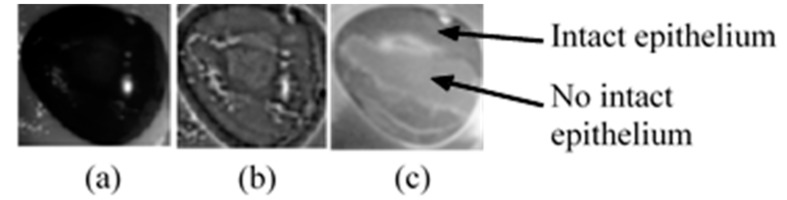
Image comparison before and after enhancement. (**a**) original without stain; (**b**) after enhancement (without stain); and (**c**) ground truth with stain.

The comparison between the image before and after enhancement is shown in Figure 16. The images show that after enhancement the boundary of the injury is visible and corresponds to the ground truth image. The image enhanced look slightly larger due to the morphological process.

**Figure 17:**
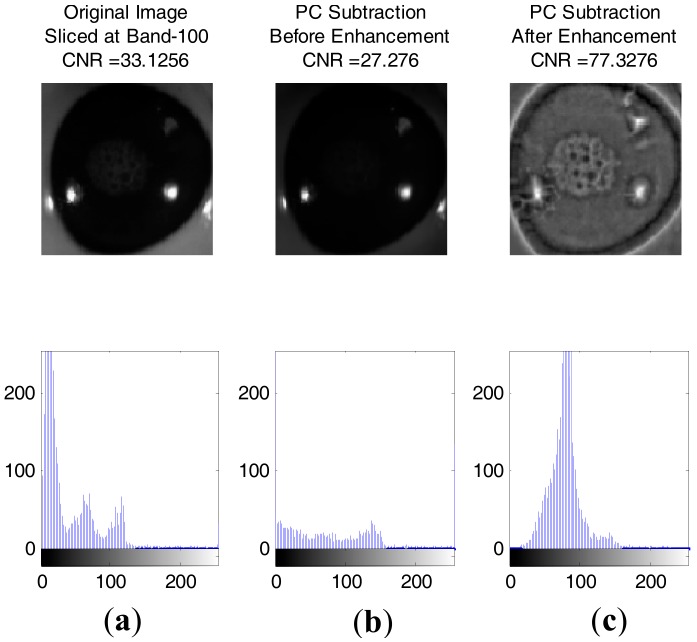
Image of healthy cornea with the histogram underneath respectively. Images were normalised and resized to 100 by 100: (**a**) Original Image sliced at band-100 (CNR: 33.1256); (**b**) Image after PC subtraction before enhancement (CNR: 27.276); (**c**) Image after PC subtraction and enhancement (CNR: 77.3276).

**Figure 18:**
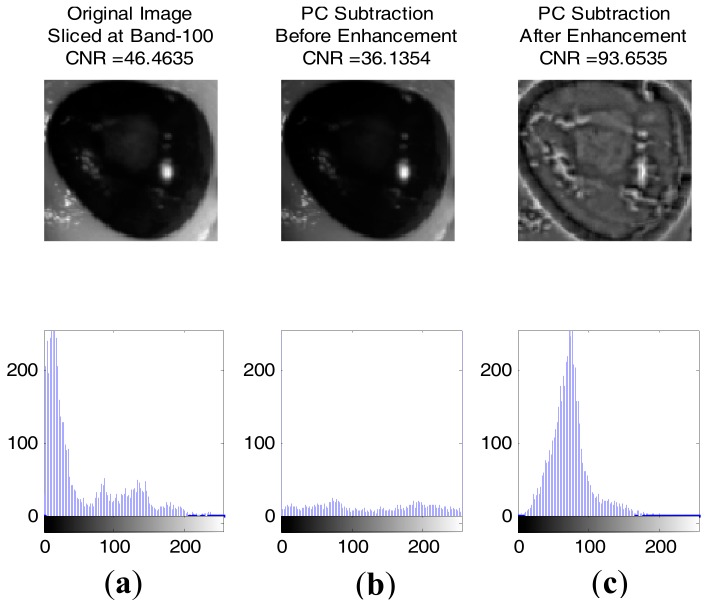
Image of injured cornea with the histogram underneath respectively. Images were normalised and resized to 100 by 100: (**a**) Original Image sliced at band-100 (CNR: 46.4635); (**b**) Image after PC subtraction before enhancement (CNR: 36.1354); (**c**) Image after PC subtraction and enhancement (CNR: 93.6535).

Figure 17 and Figure 18 show some result of images with contrast to noise ratio (CNR) [43] and the histogram between healthy and injured corneas. It is shown that the CNR value for an enhanced image is higher than the original image, making the injury easily detectable with human vision. Otherwise, the lower CNR is making the injury more difficult to detect.

Based on the histogram of the gray-scale image following the enhancement process, four features were extracted, namely mean, standard deviation (square root of the variance), skewness, and kurtosis. The results are shown in Table 3. These were calculated by using the probability distribution of the intensity levels in the histogram bins [56]. The histogram of intensity levels is a simple summary of the statistical information of the image, and individual pixels are used to calculate the gray-level histogram. Therefore, the histogram contains first-order statistical (central moments) information about the image values [57]. These statistics are defined by the following equations [58].

Let random variable I represents the gray-levels of image values. The first-order histogram P(I) is defined as:

(7) $$P\left(I\right)=\frac{No.\;of\;pixels\;with\;gray\;level\;I}{Total\;No.\;of\;pixels\;in\;the\;histogram}$$

Based on the definition of P(I), the mean and central moments µ_k_ of I given by:

Mean:

(8) $$Pm1=\overset{N-1}{\underset{I=0}{\sum }}I^{1}P\left(I\right)$$

Central moments:

(9) $${µ}_{k}=\overset{N-1}{\underset{I=0}{\sum }}{\left(I-m1\right)}^{k}P\left(I\right)$$

where k = 2, 3, 4, and N is the number of possible gray levels.

The most frequently used central moments are variance, skewness and kurtosis given by ${µ}_{2}$, ${µ}_{3}$, and ${µ}_{4}$ respectively [58]. The variance measures the deviation of gray-levels from the mean. Skewness is an indicator of asymmetry around the mean whilst kurtosis is a function of the histogram sharpness. Combinations of 2D-features were computed for both healthy and injured eyes. These features were used as inputs to the binary classifiers. Table 4 shows the results of classification with SVM-GRBF using four features extracted from the histogram for testing data (data were unseen during training). It represents the number of iterations required for convergence, the accuracy, and the error during testing for different sets of hyperparameters.

**Figure 19:**
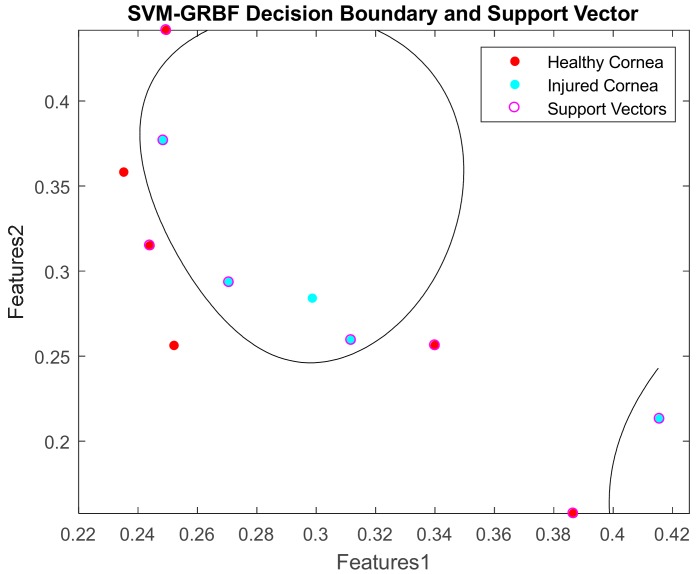
Decision boundary and support vector for Mean vs Skewness (testing data).

It was computed using LibSVM-MATLAB. The accuracy was calculated as the area under the receiver operating characteristic (ROC) curve. The error refers to the generalisation error which is the out-of-sample mean squared error. It measures how accurately a model is able to predict outcome values for previously unseen data. The result shows that a combination of 2D features of mean and skewness can yield 100% accuracy when C and Sigma are increased sufficiently. Its decision boundaries and support vectors are illustrated in Figure 19.

**Figure 20:**
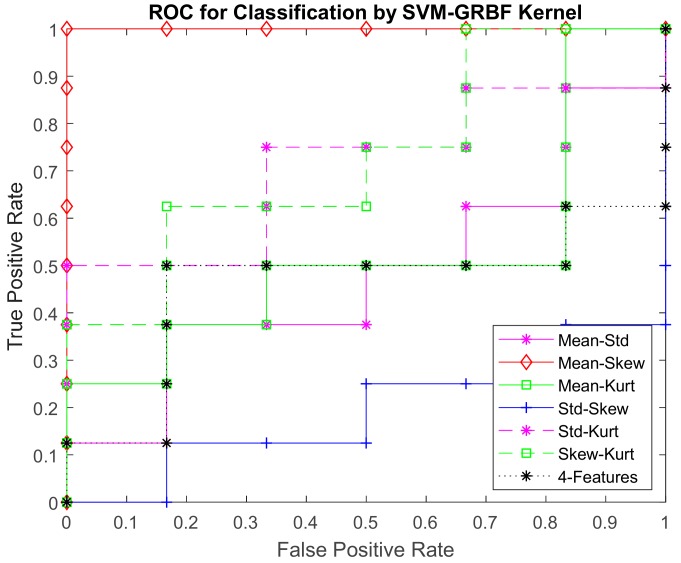
ROC curve for 2D-features classification by SVM-GRBF with C = 500, and Sigma = 1.658.

The performance of the classifier is measured using a confusion matrix as in Table 5. The ROC curve for 2D feature classification is shown in Figure 20.

The ROC is a metric used to check the accuracy of classifiers. By definition [59,60], a ROC curve shows True Positive Rate (TPR) versus False Positive Rate (FPR) for different thresholds of the classifier output. The maximum area under the curve (AUC) is 1, which corresponds to a perfect classifier. Larger AUC values indicate better classifier performance. From the ROC curve, 2D features of mean vs skewness yielded an optimal accuracy compared to other combinations of features. This ROC curve can be used for feature selection to classify cornea images.

**Figure 21:**
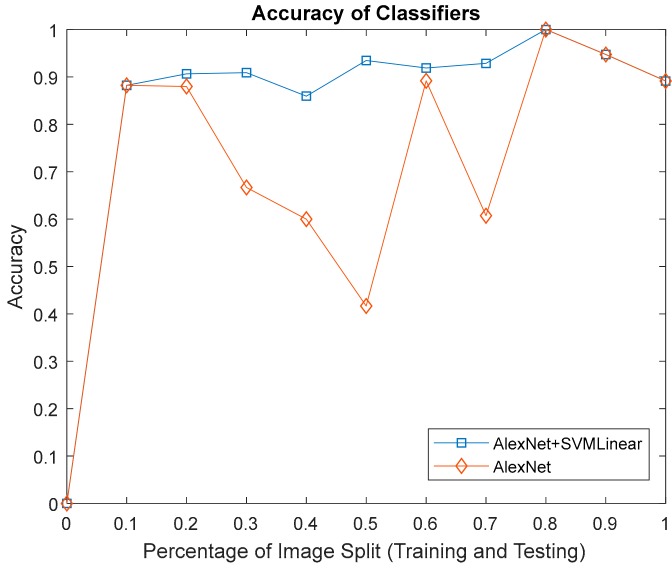
The accuracy of AlexNet and AlexNet-SVM classifier.

**Figure 22:**
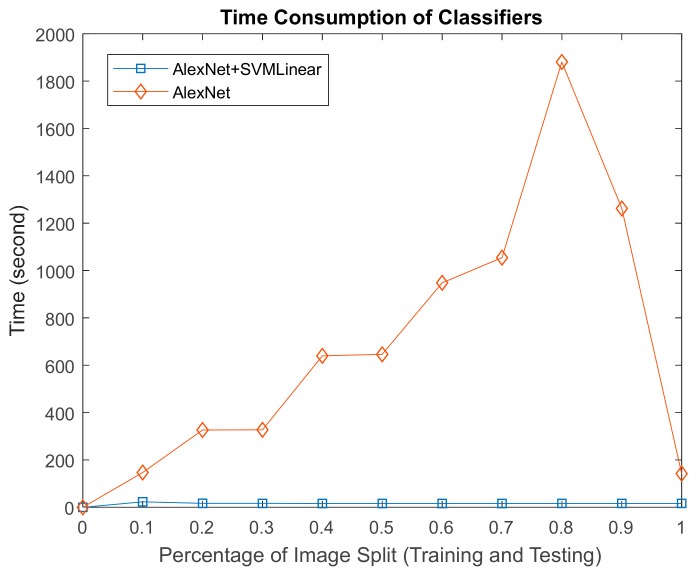
The time consumption of AlexNet and AlexNet + SVM-linear classifiers.

The results of the second and third approaches to classify physical images features according to AlexNet and AlexNet-SVM linear, with respect to accuracy as in Figure 21 and the time consumed in Figure 22. Both yielded 100% accuracy at 0.8 (80% training, 20% testing) image distribution, although AlexNet performed poorly in-terms of computation time.

## 5. Conclusions

In conclusion, the uniqueness of the cornea with five layer tissues underneath lies mainly in its transparency. This property in itself poses a huge challenge to clinicians due to changes, which are often fine and subtle and serve as a barrier to diagnosis. Therefore, the combination of hyperspectral imaging and image processing techniques has the potential to become a viable alternative solution in the assessment of corneal epithelium injury without the need for traditional contacting methods. When tested with the three classification approaches it showed promising results particularly when AlexNet was combined with SVM-linear in terms of accuracy and time. Future developments in this field could result in this work being translated for human use.

## Figures and Tables

**Table 1:** List of experimental works.

Lab	Quantity	Camera Type	Image Scanned	Remarks
Lab 1	Supplier A 5 Pigs Eyes	VIS-NIR (400 to 1000 nm)	6 Scanned (3 injured 3 healthy)	Pilot test [34] Image Dimension after binning: 1200 to 1300 × 804 × 604 302 spectral bands.
Lab 2	Supplier A 30 Pigs Eyes	VIS-NIR (400 to 1000 nm)	17 Scanned (from 8 Eyes) (5 injured + 7 Stained 1 Healthy + 1 Stained 3 No Intact Epithelium) 22 Eyes Rejected	Apply stains Image Dimension after binning: 500 to 700 × 336 × 256 256 spectral bands
Lab 3	Supplier B 12 Pigs Eyes	VIS-NIR (400 to 1000 nm)	26 Scanned (8 injured + 10 stained 4 healthy + 4 stained)	Apply stains Image Dimension after binning: 250 to 400 × 336 × 256 256 spectral bands

**Table 2:** AlexNet parameters with fine-tuned network for transfer learning on cornea images.

No	Layer	Type	Parameters
1	Data	Image Input	Layer1: Convolution layer **Input image size: 227 × 227 × 3** with zero centre normalisation No. of filters: 96 Filter size: 11 × 11 × 3 Stride: [4 4] Output: 224/4 × 224/4 × 96 (because of stride 4) **Train Network with a CPU**
2	Conv1	Convolution	
3	Relu1	Relu	Rectified linear units
4	Norm1	Cross channel normalisation	Cross channel normalisation with 5 channels per element
5	Pool1	Max pooling	Layer2: Max pooling followed by convolution Input: 55 × 55 × 96 Max pooling: 55/2 × 55/2 × 96 = 27 × 27 × 96 No. of filters: 256 Filter size: 5 × 5 × 48 Stride: [2 2] Output: 27 × 27 × 256 **Train Network with a CPU**
6	Conv2	Convolution	
7	Relu2	Relu	Rectified linear units
8	Norm2	Cross channel normalisation	Cross channel normalisation with 5 channels per element
9	Pool2	Max pooling	Layer3: Max pooling followed by convolution Input: 27 × 27 × 256 Max pooling: 27/2 × 27/2 × 256 = 13 × 13 × 256 No. of filters: 384 Filter size: 3 × 3 × 256 Stride: [2 2] Output: 13 × 13 × 384 **Train Network with a CPU**
10	Conv3	Convolution	
11	Relu3	Relu	Rectified linear units
12	Conv4	Convolution	Layer4: Convolution layer Input: 13 × 13 × 192No. of filters: 384 Filter size: 3 × 3 × 192 Stride: [1 1] Output: 13 × 13 × 384 **Train Network with a CPU**
13	Relu4	Relu	Rectified linear units
14	Conv5	Convolution	Layer5: Convolution layer Input: 13 × 13 × 192 No. of filters: 256 Filter size: 3 × 3 × 192 Stride: [1 1] Output: 13 × 13 × 256 **Train Network with a CPU**
15	Relu5	Relu	Rectified linear units
16	Pool5	Max pooling	3 × 3 max pooling with stride [2 2]
17	Fc6	Fully connected	Layer6: Fully connected layer Input: 13 × 13 × 128 is transformed into a vector Output: 4096-dimensional feature with 2048 in each vector
18	Relu6	Relu	Rectified linear units
19	Drop6	Dropout	Reducing overfitting with probability 0.5
20	Fc7	Fully connected	Layer7: Fully connected layer 4096-dimensional feature with 2048 in each vector
21	Relu7	Relu	Rectified linear units
22	Drop7	Dropout	Reducing overfitting with probability 0.5
23	Fc8	Fully connected	Layer8: Fully connected layer **2 number of classes**
24	Prob	SoftMax	Reducing overfitting
25	Output	Classification output	**Classify 2 image: Healthy and Injured**

**Table 3:** 4-features computed from image histogram for 25 eyes.

EYE Healthy	Mean Healthy	Standard Deviation Healthy	Skewness Healthy	Kurtosis Healthy	EYE Injured	Mean Injured	Standard Deviation Injured	Skewness Injured	Kurtosis Injured
1	135.31	28.10	0.69	4.79	12	125.85	26.46	0.51	5.36
2	97.23	28.67	0.92	5.86	13	110.69	26.74	0.83	6.45
3	101.50	27.87	0.84	6.09	14	81.23	22.58	1.43	8.40
4	80.91	22.34	1.22	8.73	15	82.07	23.08	0.83	5.81
5	88.11	25.90	0.95	7.26	16	76.56	19.28	1.16	8.80
6	102.41	26.99	1.04	6.47	17	79.44	28.30	1.02	5.54
7	100.73	19.88	1.10	7.74	18	67.84	20.04	1.11	8.01
8	108.48	21.03	1.25	9.66	19	73.76	27.27	1.11	6.24
9	89.85	24.00	1.26	8.21	20	116.46	27.40	0.61	4.64
10	99.75	27.72	0.89	6.08	21	120.36	21.74	0.76	6.61
11	98.96	22.73	1.18	8.66	22	96.72	27.15	0.94	6.51
					23	108.97	28.55	0.67	5.71
					24	101.93	29.25	0.27	4.67
					25	105.74	24.04	0.37	4.67

**Table 4:** Four features classification using SVM-GRBF.

Features	C = 1			C = 500			C = 500		
	Sigma = 1			Sigma = 1.658			Sigma = 2.658		
	10-Fold Cross Validation			10-Fold Cross Validation			10-Fold Cross Validation		
	Iterations	Accuracy	Error	Iterations	Accuracy	Error	Iterations	Accuracy	Error
**Mean-Std.**	13	0.2708	0.4545	81	0.5625	0.3636	578	0.4792	0.4545
**Mean-Skew**	13	0.8333	0.3636	148	0.9583	0.4545	412	1	0.4545
**Mean-Kurt**	6	0.7500	0.3636	169	0.8125	0.3636	189	0.5208	0.2727
**Std.-Skew**	10	0.6042	0.4545	161	0.2083	0.5455	207	0.1875	0.6364
**Std.-Kurt**	6	0.3750	0.1818	419	0.6875	0.6364	172	0.7083	0.4545
**Skew-Kurt**	12	0.6875	0	200	0.5833	0.1818	243	0.7292	0.0909
**4-Features**	11	0.4375	0.3636	38	0.7292	0.4545	86	0.4583	0.4545

**Table 5:** Confusion matrix.

Confusion	Predict	Predict
Matrix	Healthy	Injured
Actual	True	False
Healthy	Negative (TN)	Positive (FP)
Actual	False	True
Injured	Negative (FN)	Positive (TP)
